# Assessment of the Impact of Media Coverage on COVID-19–Related Google Trends Data: Infodemiology Study

**DOI:** 10.2196/19611

**Published:** 2020-08-10

**Authors:** Bernardo Sousa-Pinto, Aram Anto, Wienia Czarlewski, Josep M Anto, João Almeida Fonseca, Jean Bousquet

**Affiliations:** 1 Department of Community Medicine, Information and Health Decision Sciences Faculty of Medicine University of Porto Porto Portugal; 2 Center for Health Technology and Services Research University of Porto Porto Portugal; 3 MASK-air Montpellier France; 4 Medical Consulting Czarlewski Levallois France; 5 Centre for Research in Environmental Epidemiology Barcelona Institute for Global Health Barcelona Spain; 6 Universitat Pompeu Fabra Barcelona Spain; 7 CIBER Epidemiología y Salud Pública Barcelona Spain; 8 Charité, Universitätsmedizin Berlin Humboldt-Universität zu Berlin Berlin Germany; 9 Comprehensive Allergy Center Department of Dermatology and Allergy Berlin Institute of Health Berlin Germany; 10 MACVIA-France Montpellier France

**Keywords:** COVID-19, infodemiology, infodemic, Google Trends, media coverage, media, coronavirus, symptom, monitoring, trend, pandemic

## Abstract

**Background:**

The influence of media coverage on web-based searches may hinder the role of Google Trends (GT) in monitoring coronavirus disease (COVID-19).

**Objective:**

The aim of this study was to assess whether COVID-19–related GT data, particularly those related to ageusia and anosmia, were primarily related to media coverage or to epidemic trends.

**Methods:**

We retrieved GT query data for searches on *coronavirus*, *cough*, *anosmia*, and *ageusia* and plotted them over a period of 5 years. In addition, we analyzed the trends of those queries for 17 countries throughout the year 2020 with a particular focus on the rises and peaks of the searches. For *anosmia* and *ageusia*, we assessed whether the respective GT data correlated with COVID-19 cases and deaths both throughout 2020 and specifically before March 16, 2020 (ie, the date when the media started reporting that these symptoms can be associated with COVID-19).

**Results:**

Over the last five years, peaks for *coronavirus* searches in GT were only observed during the winter of 2020. Rises and peaks in *coronavirus* searches appeared at similar times in the 17 different assessed countries irrespective of their epidemic situations. In 15 of these countries, rises in *anosmia* and *ageusia* searches occurred in the same week or 1 week after they were identified in the media as symptoms of COVID-19. When data prior to March 16, 2020 were analyzed, *anosmia* and *ageusia* GT data were found to have variable correlations with COVID-19 cases and deaths in the different countries.

**Conclusions:**

Our results indicate that COVID-19–related GT data are more closely related to media coverage than to epidemic trends.

## Introduction

Infodemiology is defined as “the science of distribution and determinants of information in an electronic medium, specifically the Internet, or in a population, with the ultimate aim to inform public health and public policy” [1,2]. This field comprises both “supply-based” and “demand-based” infodemiology, with the latter assessing individuals’ health-seeking behavior (eg, through online searches) [2]. Over the years, infodemiological studies have become increasingly popular, focusing on different fields such as chronic diseases, risk behaviors, and infectious diseases [3,4]. Regarding the latter, the use of search query data to predict or monitor infectious outbreaks can be traced to back to the 2002 severe acute respiratory syndrome (SARS) epidemic [5]. Subsequent studies have been conducted on influenza and other infectious diseases. Google Trends (GT) is one of the most commonly used data sources, albeit with mixed results. In fact, despite the initial optimism regarding the use of GT for influenza prediction (Google Flu Trends) [6] and despite the strong correlation of data with influenza-related emergency department visits [7], the unsatisfactory performance of Google Flu Trends led to its discontinuation [8].

In the context of the coronavirus disease (COVID-19) pandemic, there has been interest in GT (or other data on web-based activity), particularly concerning the potential role of these data in defining the proper timing and location for practicing appropriate risk communication strategies to affected populations [9]. In Europe, significant correlations were observed between COVID-19 cases and deaths and online interest on this topic [10]. In addition, GT data were found to predict COVID-19 incidence in Iran [11]. In contrast, as the number of COVID-19 cases increased, interest in telehealth and telemedicine among the US population did not correlate with the proportion of hospitals providing telehealth services [12].

Using GT to obtain information regarding COVID-19, presents two difficulties. One is that information demand may be disproportionate to the epidemiologic on account of media coverage (as described in other contexts [5]), and the other is the low specificity of the main COVID-19 symptoms. However, regarding the latter, while cough, fever and dyspnea can also occur in several other diseases, some more specific manifestations of COVID-19 have been described. Two symptoms that appear to be more specific are anosmia and ageusia [13]. This was not widely known to the general public before the publication of an interview with Hendrik Streeck in the German newspaper *Frankfurter Allgemeine Zeitung* on March 16, 2020 [14], which was then cited by media worldwide. The identification of these more specific symptoms raised interest in whether GT data for these manifestations could better correlate with COVID-19 incidence and deaths than data for less specific symptoms. While strong correlations between searches for smell-related information and the number of COVID-19 cases and deaths have been described in several countries [15], the role of media coverage in motivating smell-related searches cannot be disregarded.

Therefore, we aimed to assess whether searches for the terms *anosmia* and *ageusia* were primarily related to media releases or to COVID-19 epidemic trends.

## Methods

This is a GT-based infodemiology study that complies with the methodological framework described by Mavragani and Ochoa [16].

### Keyword Selection

In this study, we retrieved GT data on the keywords *coronavirus* (as a virus and search term), *cough* (as a topic), *anosmia* (as a disease), and *ageusia* (as a topic).

With the exception of *coronavirus*, no other nontopic or nondisease search terms were used. In fact, we tested the search terms *loss of smell*, *hyposmia*, *olfaction*, *dysgeusia*, and *loss of taste* [8] using translations of the terms into native languages of the studied countries (using double quotation marks when searching for keywords containing more than one word); however, the data retrieved with these queries were not consistent or of sufficient quality.

### Region and Period Selection

We obtained country-level GT data for all analyses except for the worldwide analysis of the last five years. We retrieved GT data for the following time periods:

- A time frame of the last five years (up to the week of April 5 to 11, 2020): This time frame allowed us to assess worldwide search spikes of selected keywords over a long-term period.
- A time frame comprising the year 2020 (ie, the period ranging from the week of January 5 to 11 to the week of April 5 to 11, 2020): This time frame allowed us to identify the search trends for selected keywords throughout the year 2020 in 17 Western countries (where search data for *anosmia* were sufficient to perform an analysis). These GT data were plotted (without performing formal correlations) alongside data on COVID-19 cases in different countries. Note that for this time frame, we retrieved data starting on January 5 (and not on the date that the first COVID-19 case was registered in each country), not only to allow between-country comparison but also because in the Western World, news coverage on SARS-CoV-2 infection started before the first confirmed cases were identified, and also because it is possible that there were COVID-19 cases in the Western World prior to the first identified cases (which may have been reflected in symptom web searches).
- A time frame ranging from the date of the first confirmed COVID-19 case in each country until March 15: This time frame allowed a closer analysis of search trends before the media started reporting that anosmia and ageusia can be symptoms of COVID-19. To assess the impact of this media coverage, we analyzed 8 different countries and correlated web searches with the respective data on COVID-19 cases for that period. Correlations with the daily number of deaths were also performed (in this case, using a time frame ranging from the date of the first death in each country until March 15, 2020).

### Search Categories

Categories and subcategories were not selected when searching for keywords.

### Data Analysis

After plotting worldwide GT data on the selected keywords for the last five years, we retrieved GT data for the year 2020 and assessed the trends of those queries in the 17 countries where searches for *anosmia* were sufficient to perform an analysis.

To further assess the impact of media coverage on COVID-19–related GT data, and to assess whether the GT data correlated with COVID-19 cases, we focused on 8 countries in different stages of the COVID-19 pandemic: France, Germany, Italy, Portugal, Spain, the United Kingdom, Brazil, and the United States. For each country, we plotted the weekly GT data for selected keywords together with weekly data on new COVID-19 cases (numbers retrieved from official sources).

Subsequently, we performed an analysis restricted to the time period prior to March 16, 2020, the date that the media started reporting that anosmia and ageusia can be symptoms of COVID-19. In fact, from that date onward, GT data could largely reflect interest in media coverage rather than searches for symptoms that patients were experiencing. Therefore, for each country, between the date of the first confirmed COVID-19 case and March 15, 2020, we assessed the correlation (by means of the Pearson correlation coefficient, *r*) between the daily average of GT for *anosmia* and *ageusia* (herein reported as *anosmia/ageusia*) and daily data on new COVID-19 cases. Similar analyses were performed for new COVID-19 deaths (in the time frame from the date of the first COVID-19 death to March 15, 2020).

To facilitate plot reading, we plotted normalized weekly data on COVID-19 cases and deaths. That is, we plotted the total number of new COVID-19 cases and deaths as percentages of the respective maximum weekly values observed during the defined time period.

## Results

**Figure 1:**
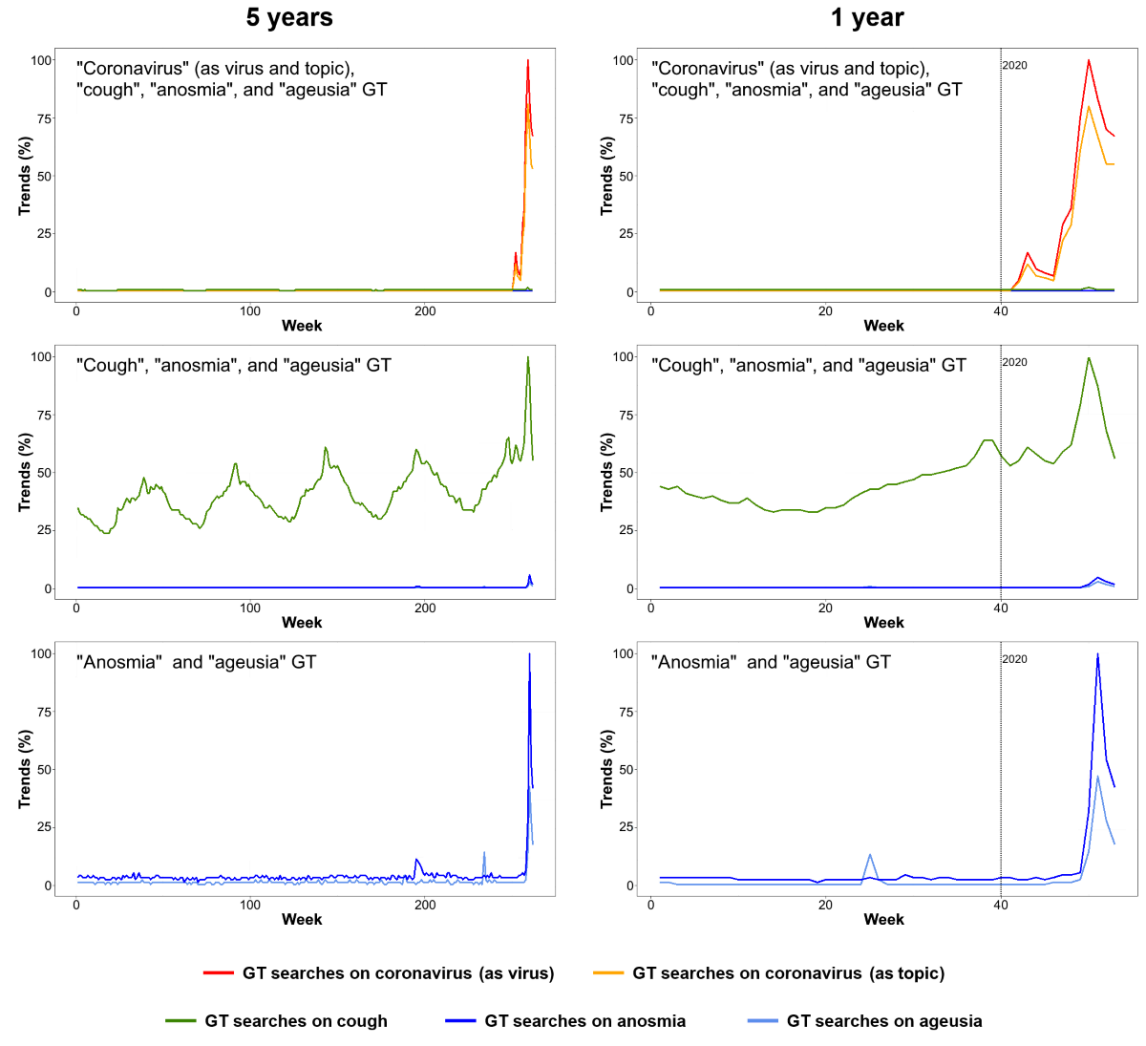
Global GT data on *coronavirus*, *cough*, *anosmia*, and *ageusia*. Data are presented as a percentage of the maximum value and on a weekly basis for periods of 5 years and 1 year up to the week of April 5 to 11, 2020. GT: Google Trends.

On a worldwide scale, the GT data for different countries showed peaks appearing at similar times, with higher peaks for *coronavirus* than for other searches (Figure 1). Throughout five years, these peaks were only found in the winter of 2020. Queries for *coronavirus* as a virus and as a search term peaked at the same time. The second highest peaks were for *cough*, with two peaks coinciding with those of *coronavirus*. *Anosmia* and *ageusia* had slightly delayed identical peaks that were not identified when the terms *cough* or *coronavirus* were searched.

We analyzed search trends in 17 countries where the *anosmia* peak was clearly identifiable (in 2 of these countries, *ageusia* did not show any peak, and in 2 others, no peak was observed for *cough*). In particular, we started by observing the week when searches for each topic started to rise (Table 1). The first rise in *coronavirus* searches started in late January 2020, while the second peak of *coronavirus* searches appeared between February 16 and 22 in one country (Italy) and between February 23 and 29 in the remaining countries. *Cough* queries started in the same week in 3 countries, 1 week later in 4 countries, 2 weeks later in 2 countries, and more than 2 weeks later in 6 countries.

**Table 1:** Weeks of onset of Google Trends peaks for search terms related to COVID-19 in 2020 in 17 countries.

Country	GT^a^ peak onset for *coronavirus* (as a virus)^b^		GT peak onset for *anosmia* (as a disease)	GT peak onset for *ageusia* (as a topic)	GT peak onset for *cough* (as a topic)
	Peak 1	Peak 2			
Argentina	January 19 to 25	February 23 to 29	March 22 to 28^c^	N/A^d^	March 8 to 14
Australia	January 19 to 25	February 23 to 29	March 22 to 28^e^	March 15 to 21^e^	February 16 to 22
Belgium	January 19 to 25	February 23 to 29	March 15 to 21^e^	March 15 to 21^e^	February 16 to 22
Brazil	January 19 to 25	February 23 to 29	March 15 to 21^e^	March 15 to 21^e^	February 16 to 22
Canada	January 19 to 25	February 23 to 29	March 15 to 21^e^	March 22 to 28^e^	March 8 to 14
Chile	January 19 to 25	February 23 to 29	March 22 to 28^c^	March 22 to 28^e^	February 23 to 29
France	January 19 to 25	February 23 to 29	March 15 to 21^e^	March 15 to 21^e^	March 1 to 7
Germany	January 19 to 25	February 23 to 29	March 15 to 21^e^	March 15 to 21^e^	February 23 to 29
Italy	January 19 to 25	February 16 to 22	March 1 to 7^e^	March 8 to 14^e^	February 23 to 29
Portugal	January 19 to 25	February 23 to 29	March 15 to 21^e^	March 15 to 21^e^	March 8 to 14
Russia	January 19 to 25	February 23 to 29	March 22 to 28^e^	N/A	N/A
Spain	January 19 to 25	February 23 to 29	March 15 to 21^e^	March 8 to 14^e^	March 8 to 14
Sweden	January 19 to 25	February 23 to 29	March 15 to 21^e^	March 22 to 28^e^	N/A
Switzerland	January 19 to 25	February 23 to 29	March 8 to 14^e^	March 15 to 21^e^	March 1 to 7
The Netherlands	January 19 to 25	February 23 to 29	March 15 to 21^e^	March 22 to 28^e^	February 23 to 29
United Kingdom	January 19 to 25	February 23 to 29	March 15 to 21^e^	March 15 to 21^e^	March 8 to 14
United States	January 19 to 25	February 23 to 29	March 22 to 28^e^	March 15 to 21^e^	March 8 to 14

^a^GT: Google Trends.

^b^Two GT peaks consistently appeared for *coronavirus*. Peak 1 is a minor peak that appeared by late January 2020, and Peak 2 is the largest Google Trends peak.

^c^GT data peaked in the week of April 5 to 11.

^d^Not applicable.

^e^GT data peaked in the week of March 15 to 21.

We observed that the onset of *anosmia* queries occurred from March 15 to 21 in 10 countries (corresponding to the week of the Hendrik Streeck interview in *Frankfurter Allgemeine Zeitung*) and from March 22 to 28 in 5 other countries (in Italy and Switzerland, the queries started before the week of March 15 to 21). The weeks of onset for *ageusia* and *anosmia* queries were the same in 7/15 countries (47%). The GT peaks for *anosmia* and *ageusia* were first observed from March 22 to 28, 2020, for all countries except Argentina and Chile (one week after the Streeck interview).

**Figure 2:**
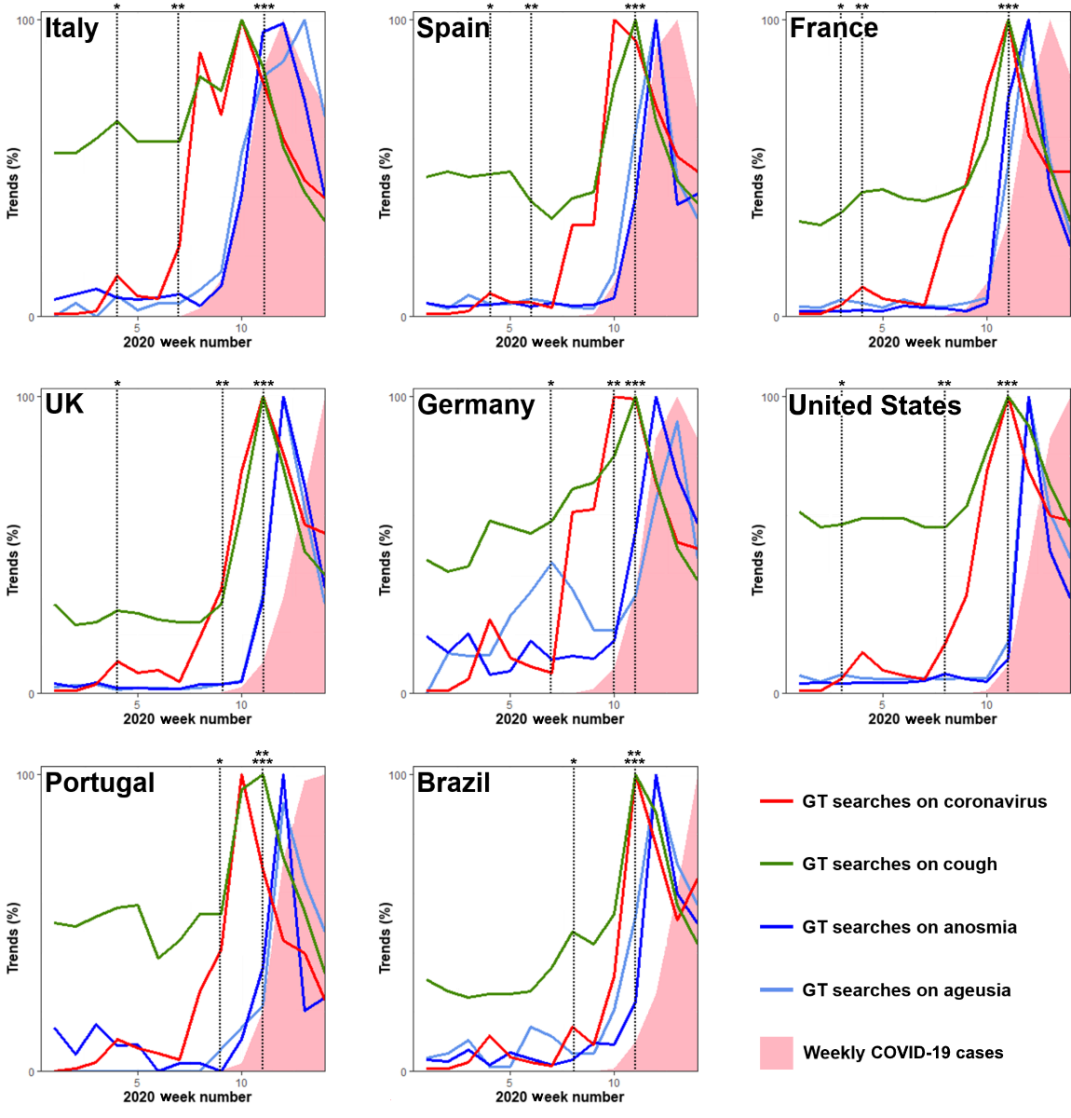
GT data for *coronavirus*, *cough*, *anosmia*, and *ageusia* and relative frequency of new COVID-19 infections. Data are presented as a percentage of the maximum value on a weekly basis, from the week of January 5 to 11, 2020, to the week of April 5 to 11, 2020. *First confirmed COVID-19 case. **First confirmed death due to COVID-19. ***Hendrik Streeck interview to Frankfurter Allgemeine Zeitung reporting that anosmia and ageusia can be COVID-19 symptoms. COVID-19: coronavirus disease. GT: Google Trends.

Subsequently, we analyzed 8 countries by plotting the average GT data for *anosmia* and *ageusia* with the number of COVID-19 cases. We observed that the GT peak coincided with the maximum weekly number of new COVID-19 cases in Italy but not in the other countries (Figure 2). For all countries (except Italy and Germany), the GT peaks were followed by sharp decreases.

**Figure 3:**
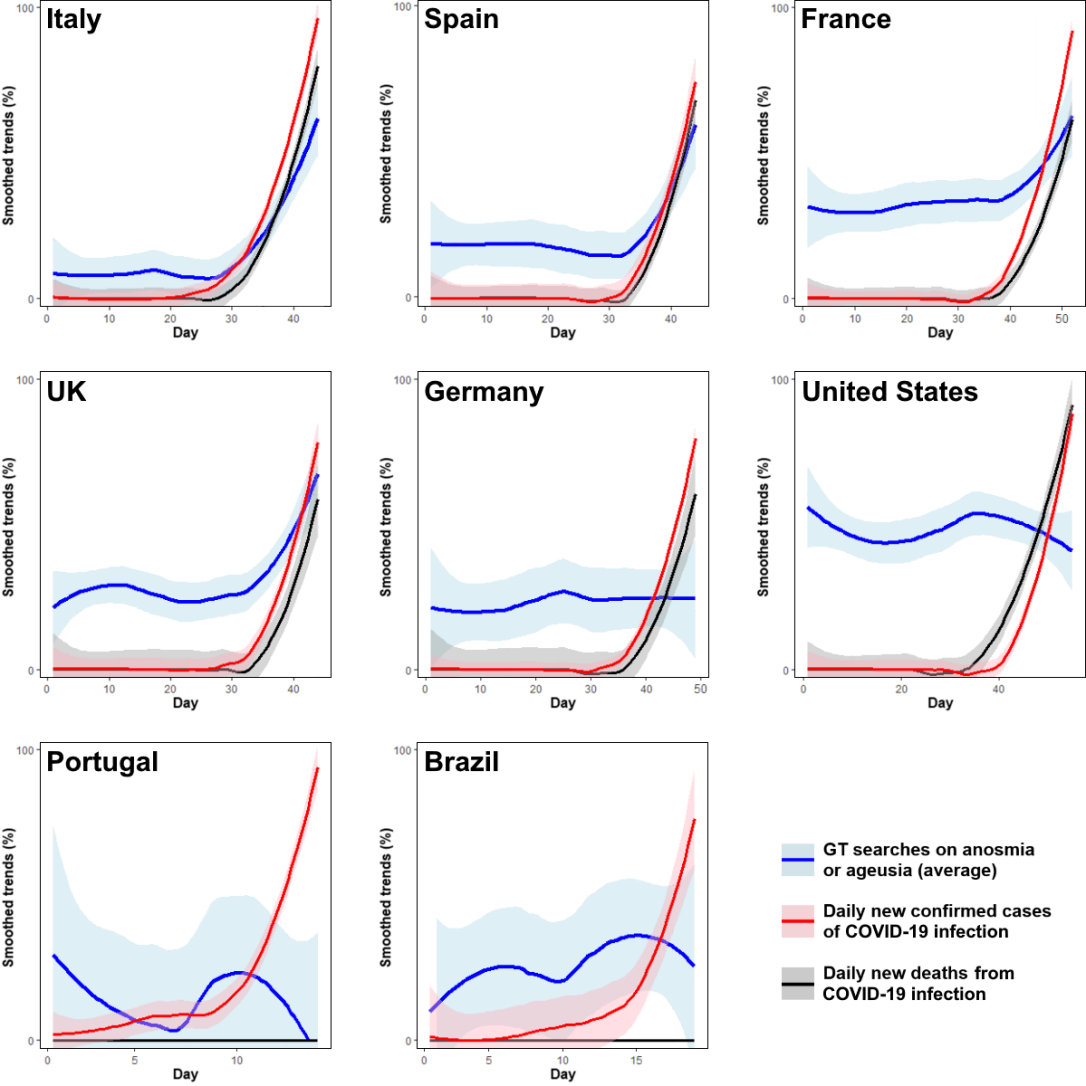
Average GT data for *anosmia* and *ageusia* and relative frequency of new COVID-19 infections and deaths. Data are presented as Loess-smoothed percentages of the maximum value (smoothed trends) on a daily basis from February 1 to March 15, 2020 (before the media publicized that anosmia and ageusia can be symptoms of COVID-19). Lines were smoothed to display trends more clearly. COVID-19: coronavirus disease. GT: Google Trends.

Analyzing the data from the date of first confirmed case of COVID-19 until March 16, we observed that in countries with higher COVID-19 infection or death rates, there were moderate to good correlations between Google Trends for *anosmia*/*ageusia* and new COVID-19 cases or deaths (Table 2, Table 3, and Figure 3). By contrast, poor correlations were observed in countries with lower COVID-19 rates by March 15. The only exception was the United Kingdom, in which we observed strong correlations between Google Trends searches on *anosmia*/*ageusia* and new COVID-19 cases (*r*=0.739) and deaths (*r*=0.668) despite the low COVID-19 infection and death rates (0.3 deaths per million inhabitants). 

**Figure 4:**
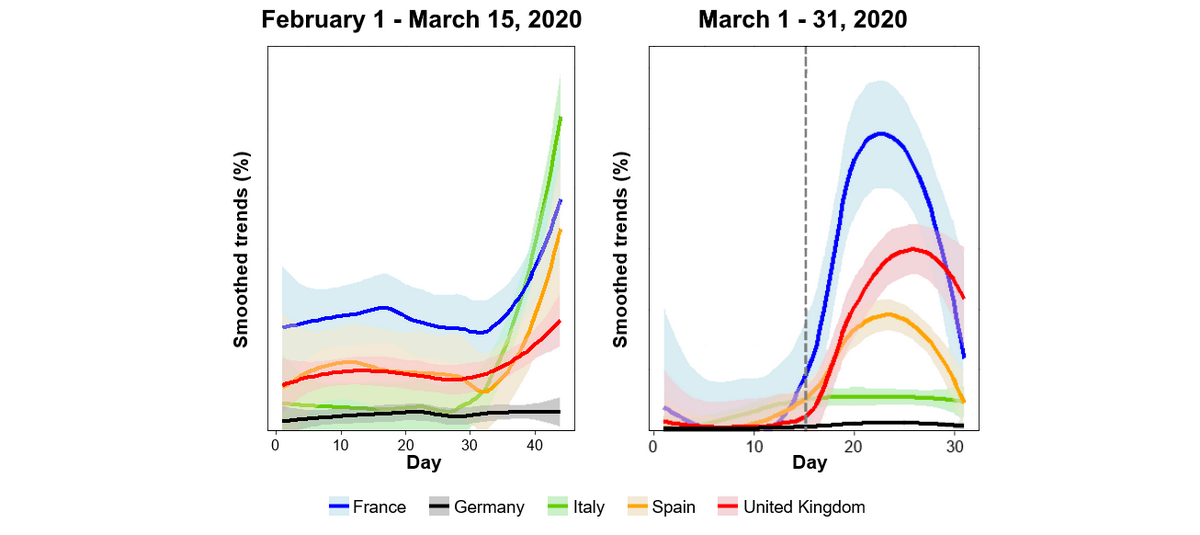
Average GT data for *anosmia* and *ageusia* before the media publicized that these terms can be symptoms of COVID-19 (February 1 to March 15, 2020) and in the 2 weeks before and after this media release (marked with a grey dashed line) (March 1 to 31, 2020). Data are presented on a daily basis as Loess-smoothed percentages of the maximum value and are adjusted for the population.

These results are supported by between-countries comparisons (Figure 4). Prior to March 16, Italy was the country with the largest volume of searches for *anosmia*/*ageusia*; however, it was surpassed by France, the United Kingdom, and Spain following extensive media coverage of those symptoms.

**Table 2:** Frequency of new COVID-19 cases and deaths in the countries examined in the study. The analysis time frame for new COVID-19 cases was from the date of the first confirmed COVID-19 case in the respective country until March 15, 2020. The analysis time frame for COVID-19 deaths was from the date of the first confirmed COVID-19 death in the respective country until March 15, 2020.

Country	COVID-19^a^ cases per million inhabitants as of March 15, 2020	COVID-19 deaths per million inhabitants as of March 15, 2020
Italy	411.1	30.0
Spain	169.6	6.24
France	80.8	1.89
United Kingdom	20.9	0.32
Germany	58.2	0.14
United States	10.6	0.19
Portugal	23.8	0
Brazil	0.95	0

^a^COVID-19: coronavirus disease.

**Table 3:** Pearson correlation coefficients between Google Trends data on anosmia/ageusia and the frequency of new COVID-19 cases and deaths in Table 2.

Country	Correlations with average GT^a^ searches for *anosmia* (as a disease)/*ageusia* (as a topic)				Correlations with GT searches for *anosmia* (as a disease)				Correlations with GT searches for *ageusia* (as a topic)			
	COVID-19^b^ cases, *r*	*P* value	COVID-19 deaths, *r*	*P* value	COVID-19 cases, *r*	*P* value	COVID-19 deaths, *r*	*P* value	COVID-19 cases, *r*	*P* value	COVID-19 deaths, *r*	*P* value
Italy	0.796	<.001	0.776	<.001	0.810	<.001	0.855	<.001	0.646	<.001	0.621	.001
Spain	0.568	<.001	0.755	<.001	0.460	.001	0.531	.002	0.506	<.001	0.632	<.001
France	0.552	<.001	0.761	<.001	0.434	.001	0.575	.008	0.438	.001	0.647	.002
United Kingdom	0.739	<.001	0.668	.025	0.663	<.001	0.745	.008	0.457	.002	0.500	.118
Germany	–0.005	.975	N/A^c^	N/A	0.104	.478	N/A	N/A	–0.099	.500	N/A	N/A
United States	–0.081	.559	–0.141	.602	0.015	.916	–0.545	.029	–0.115	.404	0.331	.210
Portugal	–0.312	.277	N/A	N/A	–0.229	.431	N/A	N/A	–0.182	.534	N/A	N/A
Brazil	–0.014	.953	N/A	N/A	–0.031	.899	N/A	N/A	0.003	.990	N/A	N/A

^a^GT: Google Trends.

^b^COVID-19: coronavirus disease.

^c^Not applicable.

## Discussion

### Principal Findings

The results of this study suggest that COVID-19–related GT queries do not necessarily follow the evolution of the epidemic and, in particular for *anosmia* and *ageusia*, are more closely related to media coverage.

Using a stepwise approach based on 1- and 5-year perspectives, we showed that search peaks not only for *coronavirus* but also for *anosmia*/*ageusia* appeared for the first time in 2020; also, there may be a relationship between the two peaks. This is different from the *cough* search term, for which searches were detected for all years but which also showed a peak coincident with the *coronavirus* peak.

We then assessed countries with an identifiable *anosmia* peak in 2020 in the northern and southern hemispheres. Surprisingly, in all countries, peaks for *coronavirus*, *cough*, and *anosmia*/*ageusia* all occurred simultaneously, irrespective of the pandemic stage. A simple interpretation is that this is unlikely to be associated with COVID-19 incidence. However, the time of onset differed for *coronavirus* or *cough* versus *anosmia* or *ageusia*; the latter coincided with the timing with which media news covered information on these symptoms (Table 4).

**Table 4:** Media coverage on the identification of anosmia and ageusia as COVID-19 symptoms.

Language	Date	Title of index media news	Source
German	March 16, 2020	*Virologe Hendrik Streeck : “Wir haben neue Symptome entdeckt”*	*Frankfurt Allgemeine Zeitung* [14]
Italian	March 17, 2020	*Coronavirus, tra i sintomi frequenti la perdita totale di gusto e olfatto*	*Corriere de la Serra* [17]
English (United Kingdom)	March 17, 2020	Coronavirus symptoms shock: Scientists discover NEW symptoms including lack of taste	*Daily Express* [18]
English (United States)	March 13, 2020	Coronavirus is most contagious before and during the first week of symptoms	*Science News* [19]
French	March 17, 2020	*Coronavirus : toux, fièvre, fatigue... quels sont les symptômes du Covid-19 ?*	*Le Parisi*e*n* [20]
Spanish	March 18, 2020	*El coronavirus neutraliza los sentidos del olfato y el gusto*	ABC [21]
Portuguese (Brazil)	March 18, 2020	*Virologista alemão revela novos sintomas do coronavírus*	*Sputnik News* [22]

We subsequently studied the peaks for *coronavirus*, *cough*, and *anosmia*/*ageusia*. The peak for *anosmia*/*ageusia* is delayed compared to that for *cough*, which is a major symptom of COVID-19. The peaks were usually short (1 week), confirming that most of the queries were driven by media coverage. Prior studies have also pointed out that GT data are highly influenced by media [23,24]; due to media coverage, aberrant ragweed pollen peaks were observed during the grass pollen season [25]. In fact, one important limitation of demand-based infodemiological studies is the difficulty of distinguishing the effects of a true biological epidemic from what generates interest or apprehension in internet users [2,5]. In that sense, complementing search data with click data has been suggested as a partial solution to overcome this limitation [2].

The correlation between *anosmia*/*ageusia* and deaths or new cases of COVID-19 varied substantially among countries. Depending on the country, there was a high correlation or no correlation at all. Prior to March 16, in countries with higher COVID-19 infection or death rates, there were moderate to good correlations between queries on *anosmia*/*ageusia* and new COVID-19 cases or deaths. This suggests that in the absence of substantial changes in media coverage and in the presence of a sufficiently high COVID-19 incidence, GT data mostly reflect searches for symptoms patients are experiencing. Thus, the strong correlations found by Walker et al [15] may reflect the facts that they analyzed GT data for *anosmia*/*ageusia* only up to March 25, 2020 (ie, up to the week before searches for *anosmia*/*ageusia* started to decrease); that their analyses on the associations between COVID-19 cases/deaths and premediatic coverage of *anosmia* GT data were restricted to three countries (the United Kingdom, Spain, and Italy); and that this premediatic coverage was considered by the authors to have occurred up to March 20, 2020 (ie, searches between March 16 and 19 were misclassified because they had already occurred under the potential influence of media coverage).

### Limitations

Our study has some potentially relevant limitations. We used data at national levels, which may have not captured within-country heterogeneity on COVID-19 incidence or GT data; different results may have been obtained if the data were assessed at a more granulated level. Another relevant limitation concerns the fact that by March 16, 2020, the incidence of COVID-19 was still low in most Western countries; with the exception of Italy and Spain, the remaining Western countries had fewer than 100 confirmed COVID-19 cases per million inhabitants. The possibility of assessing a larger number of countries with higher numbers of COVID-19 cases would have allowed us to more confidently assess *anosmia* and *ageusia* search patterns (and their association with COVID-19 epidemiology) before and after media coverage on those symptoms.

Another important GT limitation concerns the representativeness of internet users [26]. Internet use is lowest among older persons, who constitute the age group with the highest COVID-19 morbidity. Finally, GT provides relative rather than absolute numbers, which may limit across-country comparisons. However, as expected, similar correlation coefficients were obtained when comparing GT data with relative or absolute numbers of COVID-19 cases/deaths.

### Conclusions

At least in the initial stages of the SARS-CoV-2 pandemic, COVID-19–related web searches may more closely reflect media coverage (and subsequent users’ interest or apprehension) than epidemiological trends. The use of Google Trends has increased dramatically in the last decade; whereas in the past, the focus had been on surveillance and monitoring, the focus of research has now shifted to forecasting changes [27]. It appears to be important to link GT with other sources of data to overcome the limitations of using search information alone.

## Abbreviations

COVID-19: coronavirus disease
GT: Google Trends
SARS: severe acute respiratory syndrome
SARS-CoV-2: severe acute respiratory syndrome coronavirus 2
